# The Isolation Hospitals Act

**Published:** 1896-11-28

**Authors:** 


### Editor's Letter-Box.

[Our Correspondents are reminded that prolixity is a great bar to publication, and that brevity of style and conciseness of statement greatly facilitate early insertion.]

### THE ISOLATION HOSPITALS ACT.

"In a Fix" writes: Will you kindly let me know through your paper whether the County Council (Glam.) have power to compel an Urban District Council to erect an isolation hospital to any particular design and arrangements. The C.C. say that after the U.D.C. have erected the hospital and buildings to their satisfaction they will contribute half the cost of the maintenance. The U.D.C. find that to carry out the requirements of the C.C. will put them to considerably more cost than they can well afford, and being a small district, they would prefer to sacrifice the offer of the C.C., and provide one on a smaller scale.

*** According to the provisions of the Isolation Hospitals Act, 1893, it would appear that the county is moved to action either by petition or on a report from their own medical officer of health. There is nowhere in the Act anything to suggest that any local authority could bar the action of the County Council by setting up a hospital of its own. When a local inquiry is made, as it must be in whatever mode the Act is set in force, the scope of the inquiry is "as to the necessity of an isolation hospital being established" [Section 6.] When a hospital district has been established, a hospital committee is to be formed by the County Council, but in the case where no contribution is made by the County Council to the funds of the hospital, such committee may consist wholly of representatives of the local area. Nevertheless, the committee shall only have such powers as to the providing a hospital "as the County Council may delegate to them" [Section 10 (2)]. But in every case the County Council shall retain to themselves the power of inspecting any such hospital. Every hospital district shall consist of one or more "local areas" [section 8] and a "local area" means "an urban sanitary district, a rural sanitary district," &c. [section 26], so that there can be no doubt that the Act applies to urban as well as rural districts. It does not, however, extend to any county borough, or, without the consent of the Council of the borough, to any borough containing a population of 10,000 persons or upwards, or to any borough containing a less population, without the like consent, unless by order of the Local Government Board. [Section 2.] It does not appear to us that the erection of what the County Council might consider an inefficient hospital would in any way prevent them from holding an inquiry on report by their medical officer, and directing an isolation hospital for such district to be established, and it must not be forgotten that, subject to appeal to the Local Government Board, the County Council makes the regulations for the election rotation and qualification of the hospital committee. —Ed. *T. H.*

